# Hyperbranched Glycopolymers of 2-(α-d-Mannopyranose) Ethyl Methacrylate and *N*,*N*’-Methylenebisacrylamide: Synthesis, Characterization and Multivalent Recognitions with Concanavalin A

**DOI:** 10.3390/polym10020171

**Published:** 2018-02-10

**Authors:** Yuangong Zhang, Bo Wang, Ye Zhang, Ying Zheng, Xin Wen, Libin Bai, Yonggang Wu

**Affiliations:** 1 College of Chemistry and Environmental Science, Hebei University, Baoding 071002, China; zhangyuangong01@163.com (Y.Z.); azhangye0817@163.com (Y.Z.); zyyzhengying@163.com (Y.Z.); zhonggou556@hbu.edu.cn (L.B.); 2 College of Chemical Engineering and Materials, Handan University, Handan 056005, China; hdxywb@126.com

**Keywords:** hyperbranched, glycopolymers, RAFT, concanavalin A, cluster glycoside effect

## Abstract

A series of novel hyperbranched poly[2-(α-d-mannopyranosyloxy) ethyl methacrylate-*co*-*N*,*N*’-methylenebisacrylamide] (HPManEMA-*co*-MBA) are synthesized via a reversible addition fragmentation polymerization (RAFT). The dosage ratios of linear and branch units are tuned to obtain different degree of branching (DB) in hyperbranched glycopolymers. The DB values are calculated according to the content of nitrogen, which are facilely determined by elemental analysis. The lectin-binding properties of HPManEMA-*co*-MBA to concanavalin A (ConA) are examined using a turbidimetric assay. The influence of defined DB value and molecular weight of HPManEMA-*co*-MBA on the clustering rate is studied. Notably, HPManEMA-*co*-MBAs display a low cytotoxicity in the MTT assay, thus are potential candidates for biomedical applications.

## 1. Introduction

Carbohydrates and their corresponding conjugated glycoproteins are key participants in a measureless array of biological functions, and can also be used as the carrier or drug in the treatment of diseases, such as cancer, cytotoxic chemotherapy or radiotherapy [1,2,3,4,5,6,7,8]. However, the monovalent interaction between the monosaccharide units and proteins is relatively weak due to their low affinity [9,10,11,12,13,14]. Compared with monosaccharide, polysaccharides being of high molecular weight show strong affinity due to the “cluster glycoside effect” [15,16,17,18]. Apart from the effect of molecular weight, different architectures and topological expressions also have important influence on recognition event. For example, multi-block glycopolymer with a degree of monomer sequence control is a potent competitive inhibitor of the HIV envelope glycoprotein gp120 by interacting with human dendritic cell-specific intercellular adhesion molecule-3-grabbing non-integrin (DC-SIGN) [19,20].

Compared with natural hyperbranched polysaccharides, synthetic hyperbranched glycopolymers possess similar structure and property in the biomedical applications, and can also be prepared via various methods [21,22,23,24], such as ring-opening polymerization [25], atom transfer radical polymerization [26] and reversible addition-fragmentation chain transfer polymerization (RAFT) [27,28]. With the combination of living radical polymerization and click chemistry, Perrier and coworkers found a new strategy to synthesize hyperbranched glycopolymers with highly degree of branching (DB) [29]. It should be noted that the DB value played an important role in the recognition event. Technologies, such as nuclear magnetic resonance (NMR), viscosity measurements and light-scattering methods, are often used to evaluate the DB value of hyperbranched polymers [30,31,32,33]. However, in some specific conditions, these technologies are difficult to use for determining the DB value of hyperbranched glycopolymers. For instance, when NMR technology is used, the signals of proton belonging to the linear and branch unit might overlap, making it difficult to distinguish them. Aware of the importance of “cluster glycoside effect”, it would be highly desired to prepare hyperbranched glycopolymers whose DB values can be determined using a facile method.

Herein, we synthesized hyperbranched glycopolymers through the copolymerization of 2-(2,3,4,6-tetra-*O*-acetyl-α-d-mannopyranosyloxy) ethyl methacrylate (AcManEMA) and *N,N*'-methylenebisacrylamide (MBA), where MBA acted as the branch unit. To avoid the cross-link reaction, the reversible-addition fragmentation chain transfer (RAFT) polymerization was employed. The DB values of the hyperbranched glycopolymers were facilely determined via elemental analysis because the nitrogen atoms are merely contained in branch units. Moreover, the obtained hyperbranched glycopolymers bearing defined DB and molecular weight were used to investigate the binding behavior of Con A. Furthermore, the resulting hyperbranched glycopolymers were studied by MTT assay to test their cytotoxicity.

## 2. Experimental Section

### 2.1. Materials

Unless otherwise specified, all chemicals were reagent grade. D-mannose, sodium methoxide (J&K, Beijing, China), Con A (MAYA-R, Zhejiang, China) and vinyl methacrylate (TCI, Shanghai, China) were used without further purification. MBA, *N*,*N*-dimethylformamide (DMF) and acetone (Tianjin Kemiou Chemical Reagent Co., Tianjin, China) were purified before use. Distilled deionized water was prepared from a Millpore Filtration System (Millpore, Bedford, MA, USA). The chain transfer agent (CTA) 4-cyano-4-{[(ethylsulfanyl)carbonothioyl]sulfanyl} pentanoic acid and AcManEMA were synthesized according to the methods reported in the literature [34,35].

### 2.2. Measurements

^1^H NMR spectra was recorded on a Bruker AVIII 600 MHz (Bruker, Rheinstetten, Germany). Number-average molecular weight (*M*_n_), weight-average molecular weight (*M*_w_), polydispersity, and [η] were obtained by 270-doul detector-Size Exclusion Chromatography (MDSEC) (Malvern Instruments Ltd., Malvern, PA, USA), equipped with RI detector, two-angle light scattering detector and viscosity detector. The columns (15 cm viscotek G2500PW×l and 15 cm Waters Wat011545) were eluted with 0.1 M NaNO_3_ solution and calibrated with Polyethylene oxide std-PEO22K. The calibration was performed at 25 °C and a flow rate of 1 mL·min^−1^. The product dissolved in 0.1 M NaNO_3_ solution, and passed through 0.2 μm filter before injection. Recycling preparative gel permeation chromatography (GPC) purifications were performed on a Shimadzu HPLC system equipped with a model SPD-20A absorbance detector, a model RID-10A differential refractometer, an in-line degasser, a model LC-6AD pump, a model CBM-20A controller and a Shodex KF-802 preparative GPC column. The samples were cycled over the column three times before separation (eluent, THF; flow rate, 3.0 mL·min^−1^) THF. The binding behavior of HPManEMA-*co*-MBA with Con A was examined by measuring the turbidity at 420 nm [36], which were performed on a Shimadzu WV-2550 spectrophotometer (Shimadzu, Kyoto, Japan). Con A was diluted to 1 mM (based on Con A tetramer) with 10 mM HEPES-buffered (pH = 7.4) saline containing 150 mM NaCl and 1 mM CaCl_2_ (hereinafter referred to as HBS). The Con A solution (2 mL) was transferred to a 1 cm cuvette. After incubation at 25 °C, 200 μL of HBS solution of HPManEMA-*co*-MBA was injected to the cuvette, in which the final concentration of ManEMA unit was 500 μM. HeLa cells were obtained from Shanghai Institutes for Biological Sciences of the Chinese Academy of Sciences (CAS). The Cell viability was used to evaluate the cytotoxicity of HPManEMA-co-MBA. In brief, HeLa cells (5 × 10^3^ cells) in 100 μL of DMEM containing 10% FCS were plated in a 96-well plate and incubated for 24 h in a humidified atmosphere of 5% CO_2_ in air at 37 °C (Sanyo, Model MCO-18AIC, Tokyo, Japan). After 100 μL of a HPManEMA-*co*-MBA in DMEM containing 10% FCS and 2% DMSO was added, the Hela cells were incubated, and the final DMSO content was 1% in all cases. Cells incubated without HPManEMA-*co*-MBA were used as an untreated control group. After 24 h incubation, MTT dye solution (20 μL, 5 mg·mL^−1^) was added to each well, and the cells were incubated for another 4 h. The cells were analyzed using a microplate spectrophotometer (BioRad Model 3550, Richmond, CA, USA) at 570 nm, and the cell survival rate was calculated by normalization with respect to the value of control group.

### 2.3. Synthesis of HPManEMA-co-MBA

Hyperbranched glycopolymers HPAcManEMA-*co*-MBA was synthesized via RAFT polymerization. In brief, AcManEMA (1 mmol, 458 mg), a certain amount of MBA as cross-linker, and CTA (0.02 mmol, 2.77 mg), were dissolved in 4 mL of DMF, and the solution was ultrasonicated until completely dissolved. Then, oxygen was removed by repeated vacuum-nitrogen cycles (three times). After the AIBN (0.01 mmol, 1.64 mg) initiator was added into the system, the polymerization was conducted at 70 °C in an oil bath for 25 h. Afterwards, the product was precipitated by dropping the solution into a large excess of diethyl ether. The precipitated polymer was separated by centrifugation, and redissolved in acetone. The solution was precipitated again. Finally, the product was dried in vacuum to get a white solid.

HPManEMA-*co*-MBA was obtained by the deprotection of acetyl in HPAcManEMA-co-MBA. The hyperbranched polymers HPAcManEMA-*co*-MBA (200 mg) were dissolved in 8 mL CH_3_Cl:CH_3_OH (1:1), under nitrogen gas atmosphere at room temperature. After 15 min, fresh CH_3_ONa (1 mL, 1 M) was added and kept for 1 h, and the white solid was precipitated from the solution. After filtration, the white solid was dissolved in water, purified with cation-exchange resins, and finally dialyzed for 5 days, lyophilized to get white water-soluble products HPManEMA-*co*-MBA. The hyperbranched glycopolymers so obtained are denoted as **S1**, **S2**, **S3** and **S4**, and the figure refers to the feed ratio of AcManEMA and MBA (100:3, 100:5, 100:10 and 100:20, respectively). The synthetic procedure of linear polymer PManEMA was the same as that of HPManEMA-*co*-MBA, but the CTA and MBA were not used.

## 3. Results and Discussion

### 3.1. Reaction Mechanism of HPAcManEMA-co-MBA

**Scheme 1:**
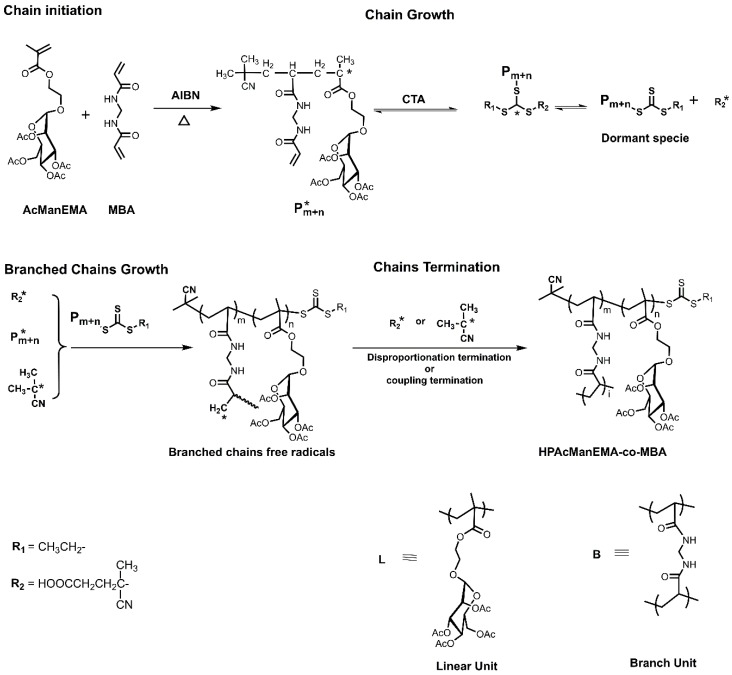
Mechanism of the polymerization of HPAcManEMA-*co*-MBA.

The mechanism for synthesis of HPAcManEMA-*co*-MBA is outlined in Scheme 1. In the initiation stage, the initiator 2,2′-azobis (2-methylpropionitrile) (AIBN) was decomposed into primary radicals, which initiated vinyl groups to obtain segments bearing radicals named chain radicals. In the chain growth stage, the RAFT polymerization mechanism illustrated that CTA reacted with chain radicals, and the new formation (dormant species) lost the ability to initiate vinyl groups. This reaction was reversible. Thus, the molecular chain would propagate until the chain radicals (P_m+n_*) or the new radicals (R_2_*) leave from the dormant species. It is worth noting that MBA contains two vinyl groups so that the monomers can easily form a network structure. Thus, in the branched chain growth stage, the other vinyl group of MBA unit was initiated by R_2_*, P_m+n_* or primary radical, forming branched chain under the help of CTA which inhibited the gelation formation [37]. In the last stage, after the oxygen was introduced in the reaction system, the chain termination was achieved by disproportionation reaction or coupling reaction.

### 3.2. Determination of DB Value

**Figure 1:**
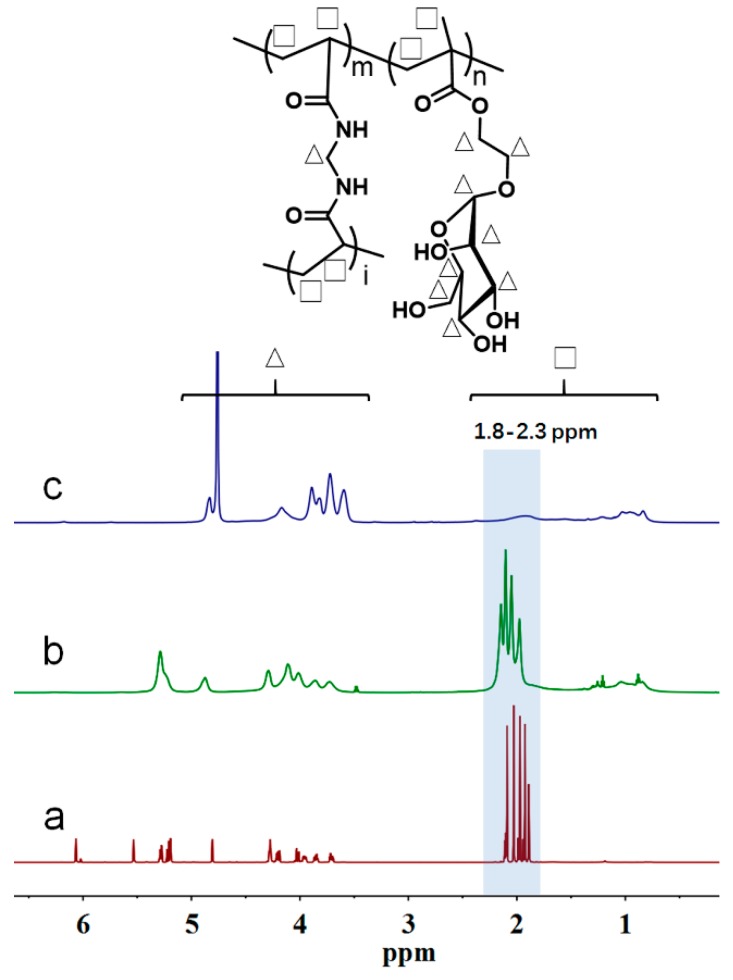
^1^H NMR spectra of: (**a**) AcManEMA in CDCl_3_; (**b**) HPAcManEMA-*co*-MBA in CDCl_3_; and (**c**) HPManEMA-*co*-MBA in D_2_O.

The structure of HPManEMA-*co*-MBA (**S1**) was characterized by ^1^H NMR. Compared with the spectrum of AcManEMA (Figure 1a), the chemical shift of HPAcManEMA-*co*-MBA (Figure 1b) was almost unchanged, but the signals of vinyl group disappeared, indicating that there was no vinyl group in the polymer structure. Meanwhile, the peaks at 0.75–1.25 ppm appeared, suggesting the formation of main chain of polymer. After the deprotection of HPAcManEMA-*co*-MBA, the peaks at 1.9–2.2 ppm, assigned to acetyl group, decreased significantly in Figure 1c, suggesting that almost all acetyl groups were removed to form HPManEMA-*co*-MBA. Therefore, the results of ^1^H NMR confirmed the successful incorporation of ManEMA and MBA units into the HPManEMA-*co*-MBA via RAFT polymerization. Nevertheless, the key parameter of DB was difficult to elucidate due to the overlapping of signals deriving from the proton in the ManEMA and MBA units.

The DB value of HPManEMA-*co*-MBA was tuned by the feed ratio of AcManEMA and MBA. The molecular weight and α value were determined by MDSEC, which was equipped with the differential refractive index (RI), viscometer, and two-angle light scattering (LS) triplet detectors. The parameters of HPManEMA-*co*-MBA (**S1**, **S2**, **S3** and **S4**), including yield, molecular weight, *α* and DB value, are shown in Table 1. The polymers showed approximately equal yields, which illustrated the activities of AcManEMA and MBA were nearly. The molecular weight increased from 8.8 × 10^4^ Dalton to 16.8 × 10^4^ Dalton with decreasing the feed ratio of AcManEMA and MBA. This might be due to the double vinyl groups of MBA that increased the probability of polymer chain re-propagating. If the resulting polymers were reinitiated from the MBA units, branch chains were formed, giving rise to a higher DB value. This surmise is also supported by the descending trend of *α* values from **S1** to **S4** in Table 1, suggesting that the corresponding DB value increased and the topological structures of HPManEMA-*co*-MBA were more compact. As presented in Table 1, the *α* values of HPManEMA-*co*-MBA were much smaller than that of linear PManEMA (0.77), which further supported the formation of hyperbranched topological structure. Nevertheless, the specific DB value was not obtained yet.

To obtain the specific DB value of HPManEMA-*co*-MBA, elemental analysis was used to measure the content of elemental C, N and H, and the results are listed in Table 2. Comparing the structure of ManEMA and MBA, elemental N was only contained in the MBA unit, thus the content of elemental N could represent the number of branch point. According to the structural formula of repeat units ManEMA (C_12_H_20_O_8_) and MBA (C_7_H_10_N_2_O_2_), the ratio of ManEMA and MBA could be calculated from Equation (1). For the units MBA, the mass ratio of C:N should be 3:1. Therefore, in Equation (1), 3N% represented the elemental C content of MBA, and the (Total C%–3N%) was the elemental C content of ManEMA. After normalization, the content ratio of ManEMA and MBA units R_(ManEMA:MBA)_ is shown in Table 2. In HPManEMA-*co*-MBA, ManEMA was the linear unit (L) and terminal unit (T), and MBA acted as branch unit (D), supported by the results of ^1^H NMR. In the low molar mass region, the number of the branch unit (D) is approximating the number of the terminal unit (T) [38], thus the DB could be written as Equation (2), and the corresponding DB values of HPManEMA-*co*-MBA were displayed in Table 2. Therefore, the results of elemental analysis were the direct evidence that DB value of samples increased from 12.4% to 30.6%.

(1) $$R_{\left(\mathrm{ManEMA}\;:\;\mathrm{MBA}\right)}=\frac{\mathrm{Total}\;C\%-3N\%}{3N\%}\times \frac{7}{12}$$

(2) $$\mathrm{DB}\%=\frac{D+T}{D+T+L}=\frac{2D}{2D+L}=\frac{2\left[\mathrm{MBA}\right]}{\left[\mathrm{MBA}\right]+\left[\mathrm{ManEMA}\right]}=\frac{2}{1+R_{\left(\mathrm{ManEMA}\;:\;\mathrm{MBA}\right)}}\times 100\%$$

### 3.3. Effect of DB and M*_n_* on The Binding Behavior between HPManEMA-co-MBA and Con A

**Figure 2:**
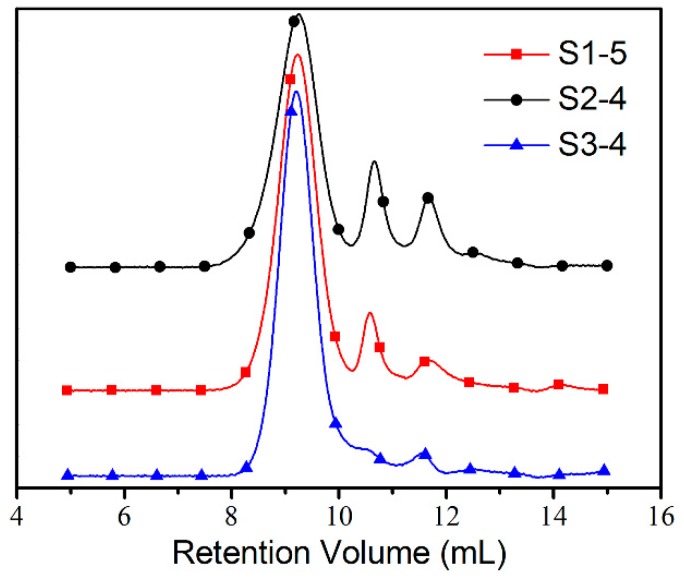
The GPC curves of the fractions (**S1-5**, **S2-4** and **S3-4**) of HPManEMA-*co*-MBA.

**Figure 3:**
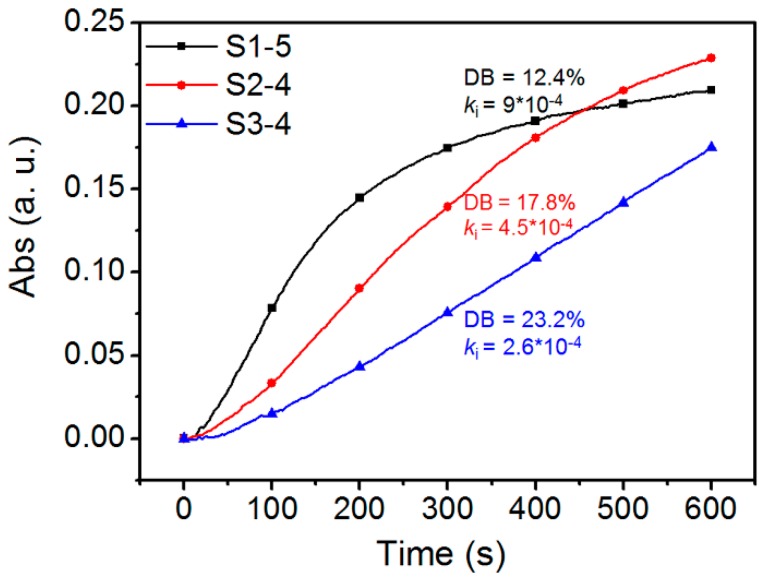
Time course of turbidity towards the binding behavior between Con A and HPManEMA-*co*-MBA (*M*_n_ = 2.7 × 10^4^ Dalton) at 25 °C. The concentration of Con A was 1 μM, and the concentration of ManEMA unit was adjusted to be 500 μM for each sample.

To obtain the influence of DB on the recognition event, HPManEMA-*co*-MBA with the same *M*_n_ but different DB values were used to investigate the binding behavior between Con A and HPManEMA-*co*-MBA. Firstly, the samples of **S1**, **S2**, **S3** and **S4** were fractionated by preparative gel permeation chromatography to yield purified HPManEMA-*co*-MBA fractions. The obtained fractions (**S1-X**, **S2-X**, **S3-X** and **S4-X**, **X** = 1, 2, 3, 4 or 5) were measured by MDSEC and labeled with *M*_n_ and α value, and the data were listed in the Table S1. As shown in Figure 2, the *M*_n_ of **S1-5**, **S2-4** and **S3-4** were nearly equal (*M*_n_ = 2.7 × 10^4^ Dalton), whereas the α values were 0.71, 0.63 and 0.54, respectively. According to Equation (2), the DB values of **S1-5**, **S2-4** and **S3-4** were 12.4%, 17.8% and 23.2%, respectively. Thus, these fractions **S1-5**, **S2-4** and **S3-4** were employed to investigate the influence of DB on the aggregation rate between Con A and HPManEMA-*co*-MBA, and the results were shown in Figure 3. The affinity of Con A and HPManEMA-*co*-MBA was evaluated in terms of the initial clustering rate (*k*_i_/ΔA_420_ s^−1^), which was obtained from the slope of the tangent at the early stage (*A*_420_ = 0.04) [39]. The corresponding *k*_i_ of **S1-5**, **S2-4** and **S3-4** were 9.0 × 10^−4^, 4.5 × 10^−4^ and 2.6 × 10^−4^, respectively, while the *k_i_* of linear PManEMA was 1.4 × 10^−2^, asreported by Obata [39]. These results revealed that the affinity of HPManEMA-*co*-MBA decreased when the DB value of HPManEMA-*co*-MBA increased from 12.4% to 23.2%. This tendency was opposite to that reported by Fernandez-Megia [40]. Note that the low epitope density of the multivalent ligand may lead to a lower rate in the initial aggregate of ligand and lectin (i.e., PManEMA and Con A) [41]. Thus, the opposite trend can be attributed to the lower content of ManEMA units in the HPManEMA-*co*-MBA.

**Figure 4:**
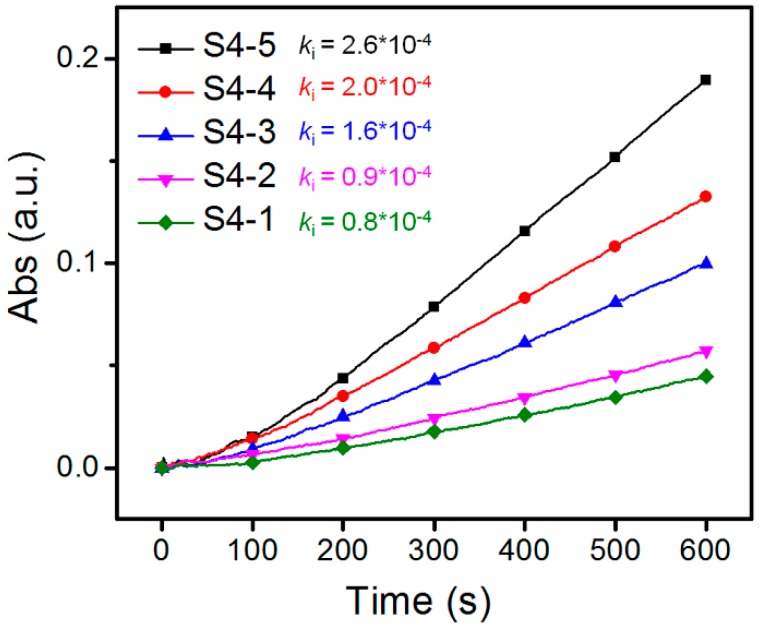
Time course of turbidity towards the binding behavior between Con A and HPManEMA-*co*-MBA (DB = 30.6%) at 25 °C. The concentration of Con A was 1 μM, and the concentration of ManEMA unit was adjusted to be 500 μM for each sample.

After sample **S4** was fractionated, the serial fractions **S4-X** (**X** = 1, 2, 3, 4, 5) would possesses the same DB value, which was supported by the results of α values (Table S1) and elemental analysis (Table S2). The *M*_n_ of fractions **S4** decreased from **S4-1** to **S4-5**, as shown in Table S1. Therefore, the serial fractions **S4-X** (**X** = 1, 2, 3, 4, 5) with the same DB (30.6%) but different *M*_n_ could be used to investigate the influence of *M*_n_ on the binding behavior between Con A and HPManEMA-*co*-MBA. As presented in Figure 4, the absorbance at 420 nm increased with decreasing of *M*_n_, and the corresponding *k*_i_ were 2.4 × 10^−4^, 2.0 × 10^−4^, 1.6 × 10^−4^, 0.9 × 10^−4^ and 0.8 × 10^−4^, respectively. As reported by Cairo [41], low epitope density of the multivalent ligand causes the low *k*_i_. The concentration of multivalent ligand can be estimated by the ManEMA unit, thus the mole concentration of HPManEMA-*co*-MBA with lower *M*_n_ should be higher than those of higher *M*_n_. Therefore, the high mole concentration means that the sample has more opportunities to form the cross-linked complexes leading to a higher *k*_i_.

### 3.4. Cytotoxicity Test of HPManEMA-co-MBA

**Figure 5:**
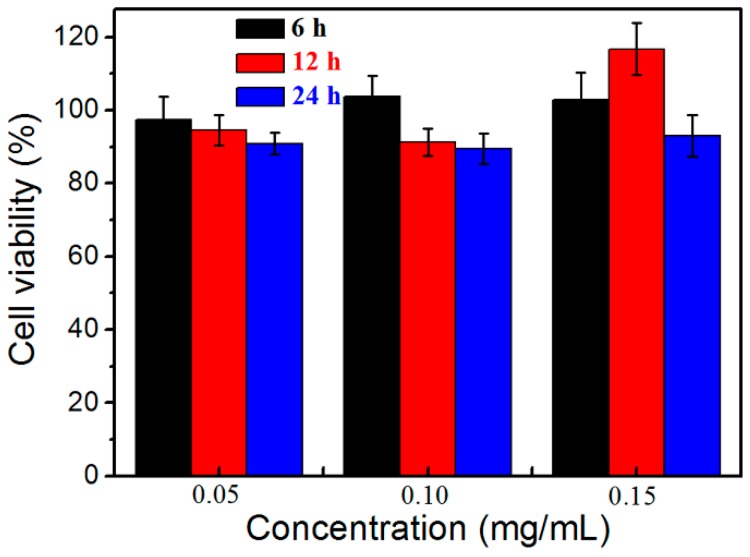
Cell viability of HPManEMA-*co*-MBA (**S4**) at different concentrations.

In the biomedical field, cell viability is often used to determine the cytotoxicity and biocompatibility of biomaterials. The cytotoxicity of HPManEMA-*co*-MBA against HeLa cells was studied using the MTT assay. Figure 5 shows the cell viability after incubation with **S4** at different concentrations. In the scope of the testing concentration, the cell viabilities were around 100% after 6 h incubation with **S4**. In the case of 0.15 mg·mL^−1^, the cell viability was higher than 90% even when the cells were incubated for 24 h. These results showed that the cytotoxicity of HPManEMA-*co*-MBA is very low in a short period of time. The good viability of obtained polymers satisfied the demand of common-used drug carriers. Owing to the low cytotoxicity, HPManEMA-*co*-MBA is proposed as a potential system for drug delivery applications.

## 4. Conclusions

In conclusion, novel hyperbranched glycopolymers HPManEMA-*co*-MBA are successfully synthesized via RAFT polymerization using the MBA as the branch unit. The DB value is facilely determined via elemental analysis because the elemental nitrogen only exists in branch units. The HPManEMA-*co*-MBA samples with the same defined DB value or molecular weight are employed to investigate the binding behavior between HPManEMA-*co*-MBA and Con A. In the case of the same *M*_n_ (*M*_n_ = 2.7 × 10^4^ Dalton) but different DB values, the clustering rate of HPManEMA-*co*-MBA and Con A decreases with the increasing of DB value. In contrast, in the case of the same DB value (30.6%) but different *M*_n_, the clustering rate increases with the decreasing of *M*_n_. In addition, the obtained HPManEMA-*co*-MBA shows a low cytotoxicity, and exhibits potential biomedical applications as drug delivery systems.

## Figures and Tables

**Table 1:** The effect of feed ratio of AcManEMA and MBA on the structure parameters. *^a,b^*.

Sample	[AcManEMA]:[MBA]	Yield%	*M*_n_ × 10^4^	*M*_w_ × 10^4^	α
**S1**	100:3	47.8	8.8	9.8	0.70
**S2**	100:5	48.2	8.5	9.6	0.63
**S3**	100:10	48.3	10.2	11.5	0.55
**S4**	100:20	58.6	16.2	20.8	0.40

*^a^* Synthesis conditions of HPAcManEMA-*co*-MBA: [AIBN]:[CTA] = 1:2, Temperature 70 °C, DMF 5 mL, Time 25 h. *^b^ M*_n_, *M*_w_ and α value were the parameters of HPManEMA-*co*-MBA.

**Table 2:** Elemental analysis of C, N, H for HPManEMA-*co*-MBA.

Elemental	C/%	N/%	H/%	R_(ManEMA:MBA)_	DB%
**S1**	44.45	0.55	6.67	15.13	12.4
**S2**	44.47	0.80	6.85	10.23	17.8
**S3**	44.29	1.06	6.81	7.62	23.2
**S4**	43.22	1.37	5.22	5.55	30.6

## Supplementary Materials

The following are available online at http://www.mdpi.com/2073-4360/10/2/171/s1. Figure S1: ^1^H NMR spectrum of AcManEMA; Figure S2: The curve of Log [η] vs. Log *M* of sample **S1**; Figure S3: The curve of Log [η] vs. Log *M* of sample **S2**; Figure S4: The curve of Log [η] vs. Log *M* of sample **S3**; Figure S5: The curve of Log [η] vs. Log *M* of sample **S4**; Table S1: The fractionation of HPManEMA-*co*-MBA; Table S2: Elemental analysis data of C, N, H of sample **S4**.
