# Supplementary material for: Hyperbranched Glycopolymers of 2-(α-d-Mannopyranose) Ethyl Methacrylate and N,N’-Methylenebisacrylamide: Synthesis, Characterization and Multivalent Recognitions with Concanavalin A
Source: Polymers (Basel). 2018 Feb 10;10(2):171. doi: 10.3390/polym10020171 (PMC6415052; doi:10.3390/polym10020171)
Supplement: Supplementary file 1 [file polymers-10-00171-s001.docx]

Supplementary Material

Hyperbranched Glycopolymers of 2-(α-D-mannopyranose) Ethyl Methacrylate and *N*,*N*’-Methylenebisacrylamide: Synthesis, Characterization and Multivalent Recognitions with Concanavalin A

Yuangong Zhang ^1^, Bo Wang ^2^, Ye Zhang ^1^, Ying Zheng ^1^, Xin Wen ^1,^*, Libin Bai ^1,2^ and Yonggang Wu ^1,^*

^1^ College of Chemistry and Environmental Science, Hebei University, Baoding 071002, China;
zhangyuangong01@163.com (Y.Z.); azhangye0817@163.com (Y.Z.); zyyzhengying@163.com (Y.Z.); zhonggou556@hbu.edu.cn (L.B.);

^2^ Handan university, Handan, 056005, China; [hdxywb@126.com](mailto:hdxywb@126.com) (B.W.)

***** Correspondence: wenxin767@hotmail.com (X.W.); wuyonggang@hbu.edu.cn (Y.W.);
Tel./Fax: +86-312-507-9317 (Y.W.)

**Table of contents**

**1**. [**Figure S1.** ^1^H NMR spectrum of AcManEMA Page S2](#_Toc477335388)

**2.** [**Figure S2.** The curve of Log *[η]* vs. Log *M* of sample S1 Page S2](#_Toc477335389)

**3.** [**Figure S3.** The curve of Log *[η]* vs. Log *M* of sample S2 Page S3](#_Toc477335390)

**4.** [**Figure S4.** The curve of Log *[η]* vs. Log *M* of sample S3 Page S3](#_Toc477335391)

**5.** [**Figure S5.** The curve of Log *[η]* vs. Log *M* of sample S4 Page S4](#_Toc477335392)

**6.** [**Table S1.** The fractionation of HPManEMA-*co*-MBA Page S4](#_Toc477335395)

**7.** [**Table S2.** Elemental analysis data of C, N, H of sample S4 Page S5](#_Toc477335394)


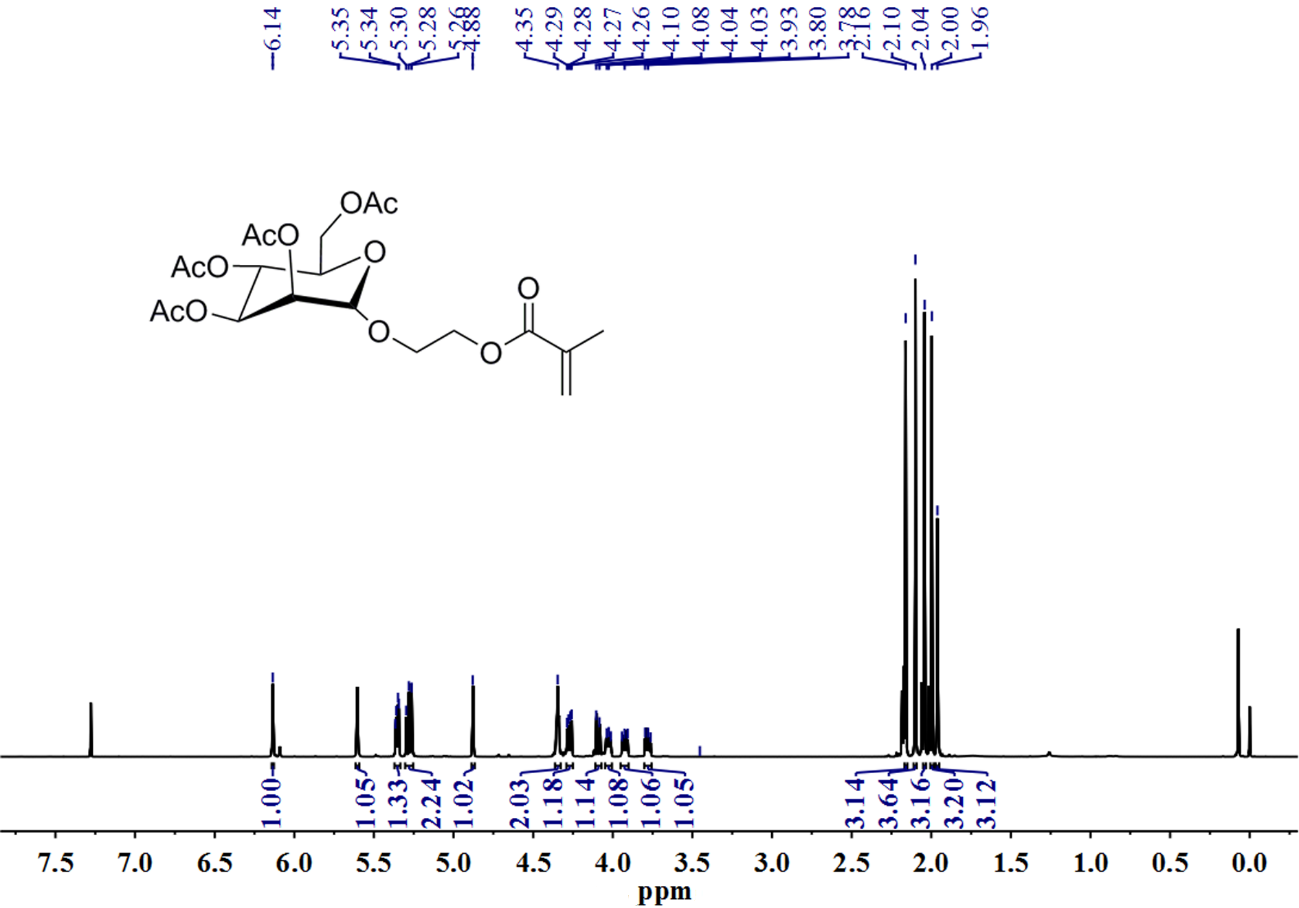


**Figure S1.** ^1^H NMR spectrum of AcManEMA


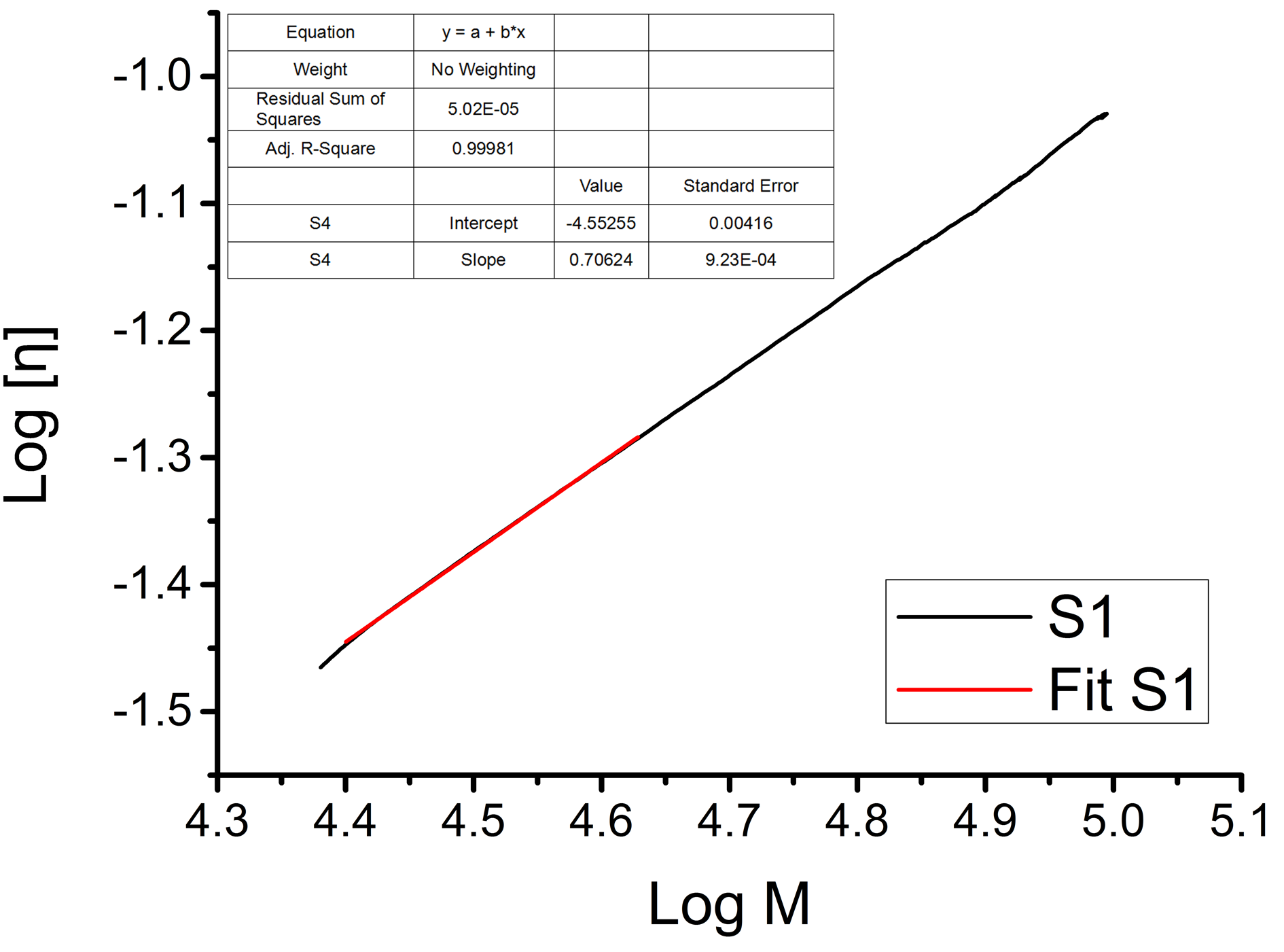


**Figure S2.** The curve of Log *[η]* vs. Log *M* of sample S1


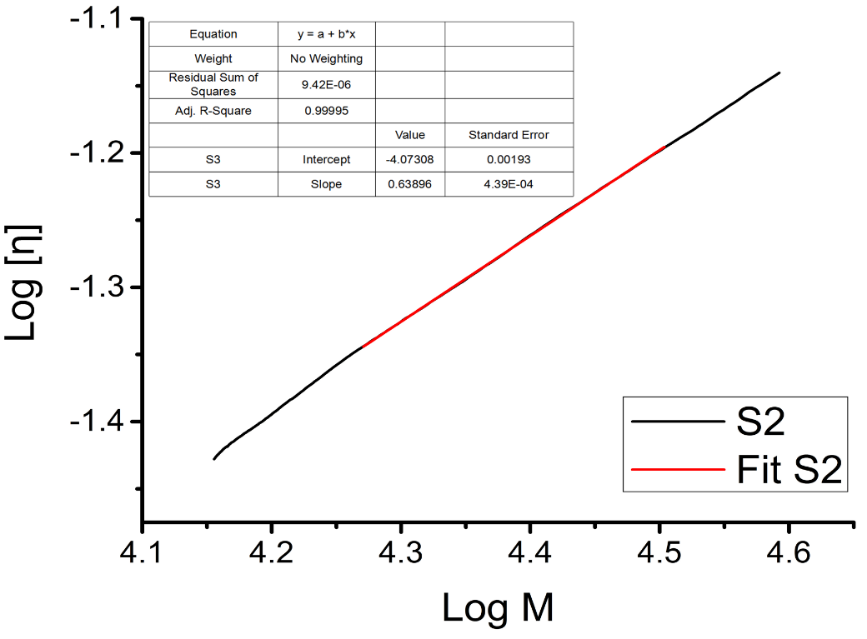


**Figure S3.** The curve of Log *[η]* vs. Log *M* of sample S2


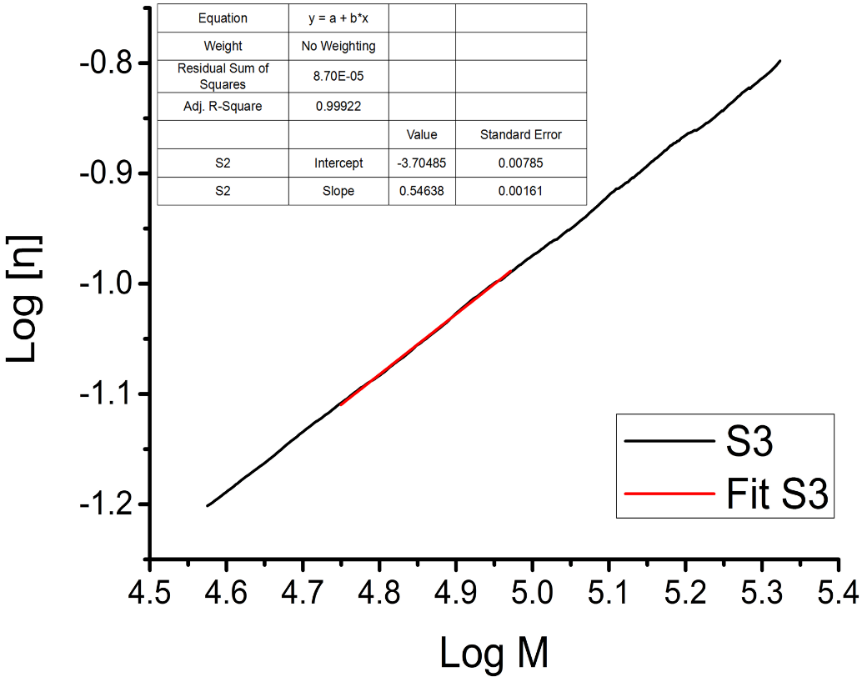


**Figure S4.** The curve of Log *[η]* vs. Log *M* of sample S3


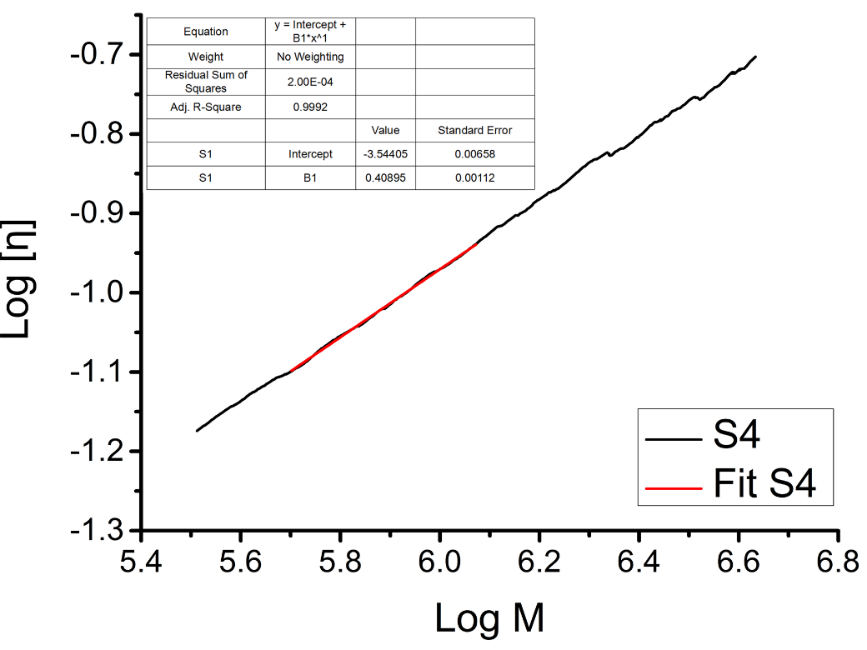


**Figure S5.** The curve of Log *[η]* vs. Log *M* of sample S4

**Table S1.** The fractionation of HPManEMA-*co*-MBA

| Sample | Fraction | *M_n_*×10^4^ | *α* |
| --- | --- | --- | --- |
| S1 | S1-1 | 24.3 | 0.70 |
|  | S1-2 | 12.5 | 0.71 |
|  | S1-3 | 4.0 | 0.70 |
|  | S1-4 | 3.1 | 0.70 |
|  | S1-5 | 2.7 | 0.71 |
| S2 | S2-1 | 25.3 | 0.63 |
|  | S2-2 | 4.8 | 0.63 |
|  | S2-3 | 4.0 | 0.62 |
|  | S2-4 | 2.7 | 0.63 |
| S3 | S3-1 | 27.0 | 0.55 |
|  | S3-2 | 6.8 | 0.54 |
|  | S3-3 | 4.3 | 0.55 |
|  | S3-4 | 2.7 | 0.54 |
| S4 | S4-1 | 29.3 | 0.39 |
|  | S4-2 | 21.1 | 0.39 |
|  | S4-3 | 18.5 | 0.40 |
|  | S4-4 | 16.9 | 0.40 |
|  | S4-5 | 8.8 | 0.40 |

**Table S2.** Elemental analysis data of C, N, H of sample S4.

| elemental | S4-3 | S4-4 | S4-5 |
| --- | --- | --- | --- |
| C/% | 43.42 | 43.06 | 43.60 |
| N/% | 1.38 | 1.35 | 1.41 |
| H/% | 5.25 | 5.03 | 5.42 |

© 2018 by the authors; licensee MDPI, Basel, Switzerland. This article is an open access article distributed under the terms and conditions of the Creative Commons by Attribution (CC-BY) license (http://creativecommons.org/licenses/by/4.0/).
